# A pictorial review of imaging manifestations of rhino-orbito-cerebral mucormycosis–emerging threat in COVID pandemic

**DOI:** 10.1186/s43055-022-00735-x

**Published:** 2022-03-04

**Authors:** Ankita Aggarwal, Nishu Raj, Krishna Bhardwaj, Ritu Nair Misra, Amita Malik, Sunil Bajaj

**Affiliations:** Department of Radiodiagnosis, VMMC and Safdarjung Hospital, Delhi, 110029 India

**Keywords:** Mucormycosis, COVID-19, Infection

## Abstract

Mucormycosis is an aggressive invasive fungal infection caused by mycocetes fungi. It is an opportunistic infection, associated with high morbidity and mortality. In the current era of COVID-19 pandemic, the entire world has witnessed a dramatic upsurge in cases of Mucormycosis. Paranasal sinuses are the commonest site to be affected with the tendency for rapid spread to orbit, face, and brain. Early diagnosis and prompt medical or surgical intervention are the only ways for preventing morbidity and saving precious lives. Imaging plays a crucial role not only in diagnosis but also in defining the extent of the disease for presurgical mapping. Black turbinate sign in the nasal cavity, mucosal thickening in paranasal sinuses with periantral invasion, and bony erosion are the early diagnostic signs. This pictorial review shall provide a comprehensive review of the various imaging manifestations of rhino-orbito-cerebral mucormycosis with a final proposed reporting checklist.

## Introduction

Mucormycosis also called zygomycetes is an invasive fungal infection caused by mycocetes fungi. They belong to the order Mucorales and the most common species to infect is Rhizopus and Mucor. These are opportunistic as they infect people with low immune status [1]. According to a recent systematic analysis conducted by Singh et al., pre-existing diabetes was the most common predisposing factor, seen in 80% of the population. Concomitant diabetic ketoacidosis was observed in 14.9% of the cases. The next common risk factor was a high intake of corticosteroids. Other less common predisposing factors included immunodeficiency, organ transplant, iron overload, post-pulmonary tuberculosis, and chronic kidney disease [2].

Mucormycosis is angioinvasive and causes ischemia with subsequent infarction and necrosis of the affected tissue. With the emergence of the COVID-19 pandemic, especially after the second wave, there has been a steady rise in cases as well as deaths due to mucormycosis. The possible pathophysiology of their association has been proposed by a few studies. As COVID-19 severely infects the lung tissue and results in the formation of multiple large alveolo interstitial lesions, Mucormycosis which is also an airborne disease has a high tendency to co-infect. Another reason is attributed to lower immunity due to reduction of CD4 and CD 8 lymphocytic counts and raised interleukin caused by SARS-associated Coronavirus (SARS-Cov) which results in opportunistic infections like Mucormycosis [3].

Rhino orbital cerebral mucormycosis can have an aggressive local spread or through the vessels (hematogenous) or perineural spread. As the disease is known to have very high morbidity and mortality, ranging over 50%[4], prompt diagnosis enables appropriate management (medical or surgical) is of prime importance. The clinical presentation of fungal sinusitis is similar to the other commoner forms of sinusitis. The patient may present with sinus pain, congestion, or hyposmia. On examination, there is erythema of the nasal mucosa along with necrotic eschars. Blood-tinged discharge from the nose and the presence of necrotic eschars should make the clinician suspicious of fungal etiology [5].

Imaging plays a pivotal role in establishing the diagnosis, looking for complications, delineating the extent of the disease, and anatomical mapping before surgical debridement. MRI is specifically useful for the assessment of the extra sinus spread [6]. Definitive diagnosis is, however, established on histopathology. We hereby present a pictorial review of the imaging manifestations of Mucormycosis affecting the paranasal sinuses, nasal cavities, orbit, infratemporal fossa, cavernous sinus, and brain, which is one of the emerging infections in the current era of COVID-19 pandemic.

## Nasal spread

**Fig. 1:**
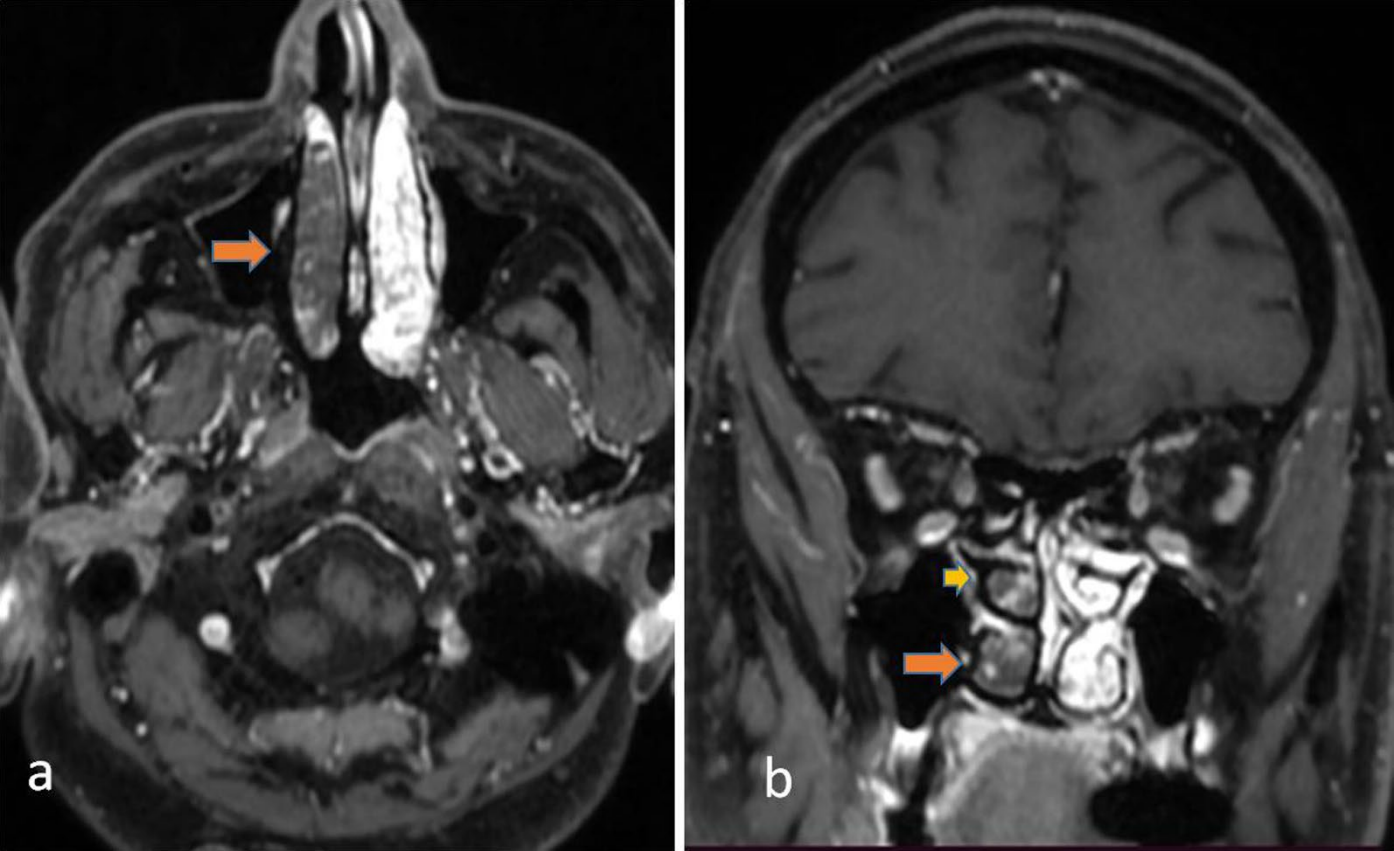
In a 60-year-old male patient having history of COVID infection 1 month back, presented with nasal discharge and headache. Contrast-enhanced axial (**a**) and coronal T1 W (**b**) images showing non enhancing right middle (arrowhead) and inferior turbinates (arrow) indicative of “*Black turbinate sign*”

Inhalation of the spores in immunocompromised patients leads to the infestation of the spores in the nasal mucosa. A weak immune response prevents the spores from getting phagocytosed and allows them to proliferate. As the infection is angioinvasive in nature, it results in ischemia and infarction of the infested tissue which appears as non-enhancing areas on Computed Tomography (CT) or Magnetic Resonance Imaging (MRI). There can be hypertrophy of the turbinates with enhancing mucosa. Non-enhancing soft tissue may be appreciated which is commonly referred to as the “black turbinate sign”[7] (Fig. 1). The septum can be involved with a high incidence of septal perforation [8].

## Paranasal sinuses

Paranasal sinuses are one of the common sites to be infected in mucormycosis. According to a study by Therakathu et al. [8], the ethmoid sinus was the most common sinus to be involved, followed by the maxillary sinus. Unilateral involvement with the sinus involvement of multiple sinuses was more frequently seen [8].

**Fig. 2:**
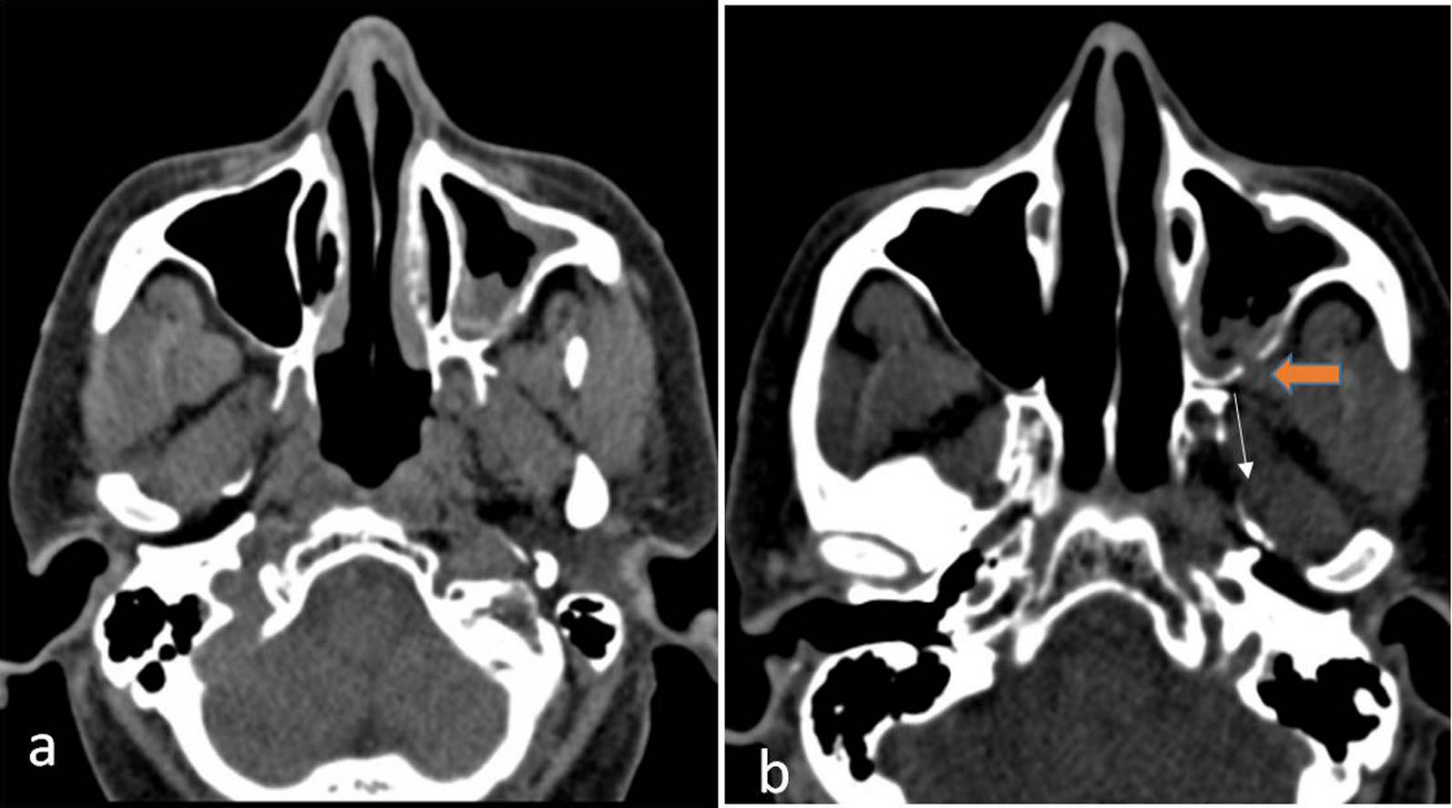
In a 35-year-old patient post COVID-infection of moderate severity, presented with sinusitis. Axial CT images (**a**, **b**) showing left maxillary sinusitis with the erosion of the posterior wall of the left maxillary sinus (orange arrow) and obliterating the retroantral fat, consistent with invasive fungal sinusitis

On imaging, in early infection, there may just be mild nodular mucosal thickening which may progress to opacification of the entire sinus. On CT, this may present as enhancing soft tissue thickening of the sinus which on MRI will be hypointense on T1 Weighted (W) and variably hyperintense on T2 W sequences. The hyphae of the fungi may show diffusion restriction. Enhancement patterns can be variable ranging from mild to heterogeneous to peripheral enhancement. Fungal infections characteristically cause erosion of the underlying bones in about 40% of the cases [9] (Fig. 2). However, this occurs late in the disease and is best detected on a CT scan. Early diagnosis can be made if enhancing soft tissue is seen anterior or posterior to the involved maxillary sinus, best detected on MRI [10]. There may also be a presence of high-density contents within the sinus which may show blooming on gradient images. These represent the fungal hyphae contents. At times, due to invasion of the smaller vessels, there may be infarction of tissue resulting in non-enhancement of the mucosa [7].

Mucosal thickening may also be seen in bacterial or viral infections of the sinuses. An acute bacterial infection usually has associated air-fluid levels, though it can be seen in fungal infection as well. Viral infection may also have non-specific mucosal thickening. Fungal sinusitis can be diagnosed if there is mucosal thickening with bony erosion with the presence of high-density contents within the sinus in the presence of an appropriate clinical setting [11, 12].

**Fig. 3:**
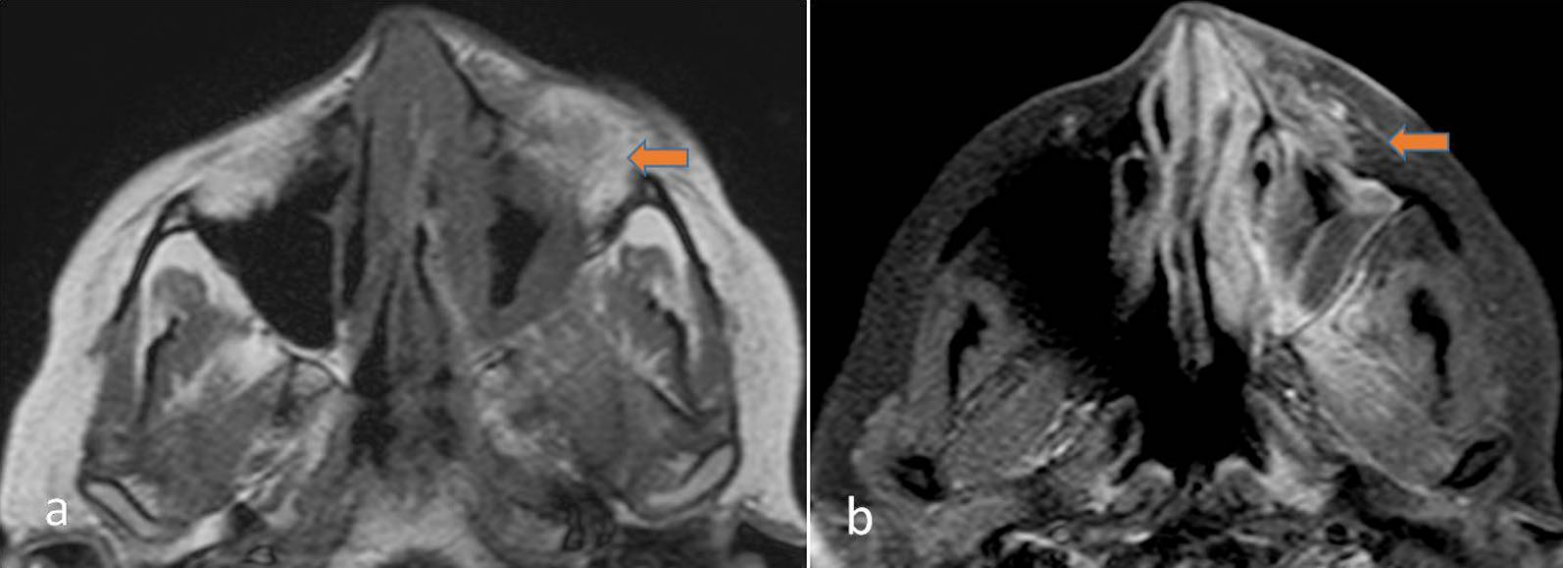
A 48-year-old male with a history of COVID 15 days back, diagnosed with Mucormycosis. T1W axial (**a**) and Post-contrast T1W (**b**) images showing left maxillary sinusitis with loss of preantral (arrow) and postantral fat plane with invasion into the infratemporal fossa

Also one should always look for the obliteration of retroantral clear fat pad. In the case of unilateral disease retroantral fat pad bilaterally must always be compared for any obliteration of fat pad (Fig. 3). Retroantral and periantral fat pad is an early sign of fungal invasion. Since fungal elements have a propensity to spread along blood vessels, obliteration of perimaxillary and retro antral fad could be the result of the perivascular spread of fungal infection beyond the confines of the maxillary sinus [10].

## Spread to infratemporal fosse/pterygopalatine fosse, masticator space

**Fig. 4:**
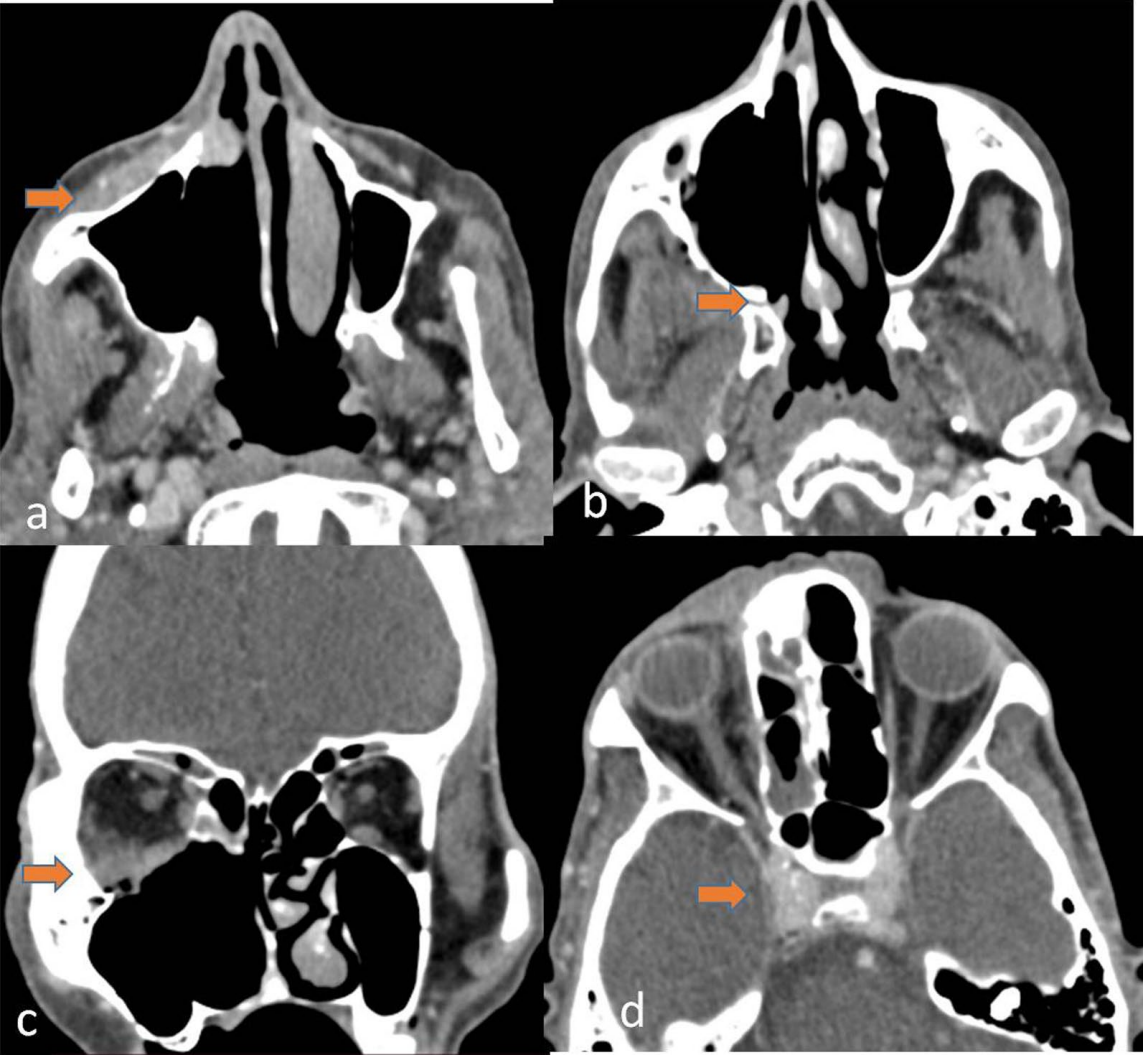
A 46-year-old male with a history of post debridement after recurrent Mucormycosis. CECT axial and coronal images depicting enhancing soft tissue with obliteration of fat in the premaxillary region (arrow) (**a**), retromaxillary region (arrow) (**b**), extension into inferior part of orbit (arrow) (**c**), and cavernous sinus (arrow) (**d**)

Mucormycosis is an aggressive infection with the tendency to invade the surrounding areas. The infection can spread to the infratemporal fossa, pterygopalatine fossa, masticator space, or premaxillary region, which will appear as soft tissue thickening along with fat stranding similar to the sinus soft tissue [8], which can be distinctly seen on CT and MRI (Figs. 3, 4).

## Spread to orbit

**Fig. 5:**
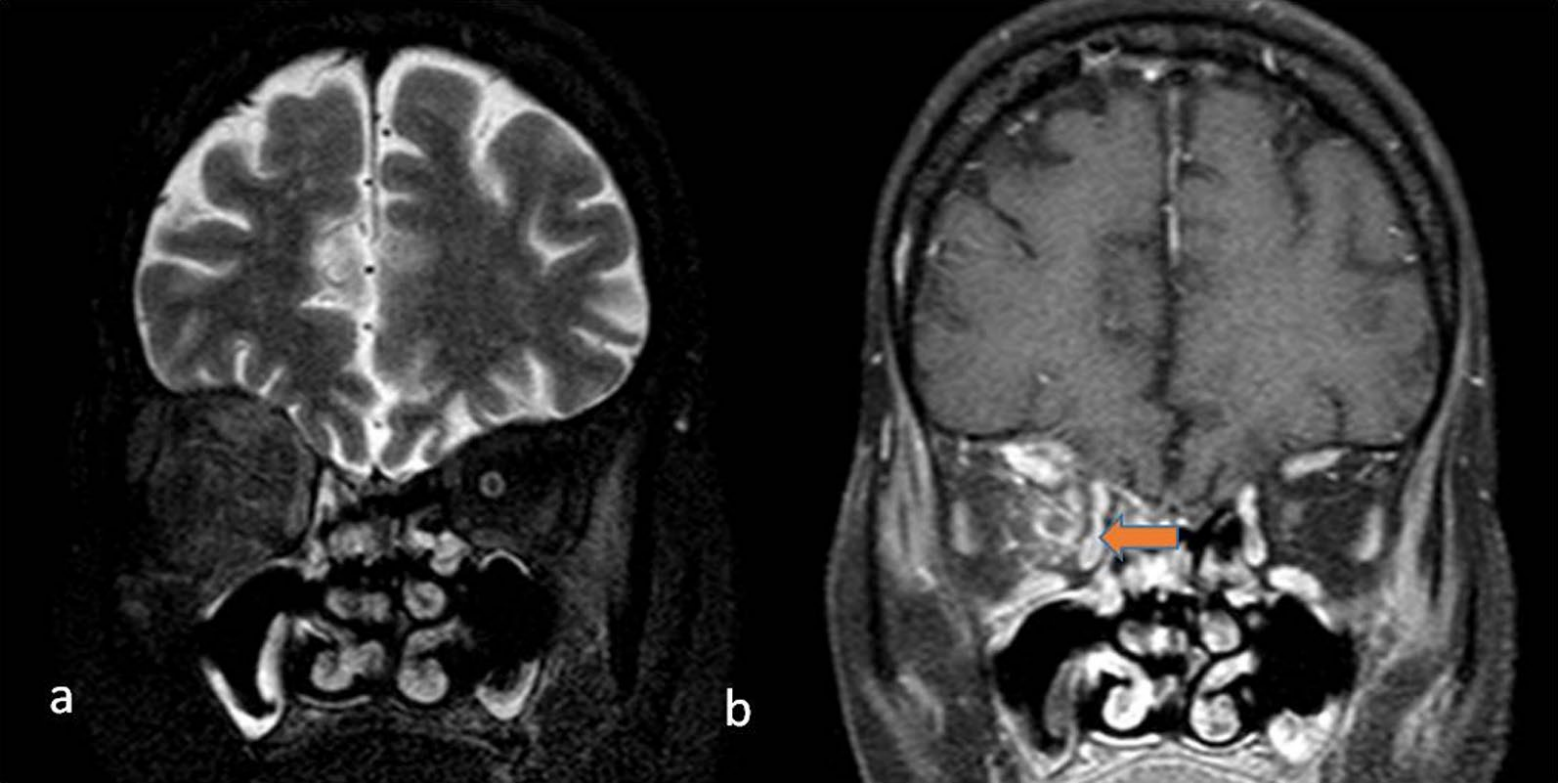
50-year-old female with a history of COVID infection 2 months back, presented with headache, right-sided proptosis, and retro-orbital pain. Coronal T2 W (**a**), Coronal T1W fat sat post-contrast (**b**) images showing extensive intraorbital fat stranding as compared to the normal intraorbital fat on the left side. Enhancement of bilateral extraocular muscles and right optic nerve is seen

Along with symptoms of sinusitis, if there is periorbital swelling, ophthalmoplegia, or proptosis, orbital invasion should be suspected. This is the most common extra sinus site to be involved in Mucormycosis [8]. Optic and infraorbital nerve invasion is suspected when there is blurred vision or facial numbness below the orbit. Mucormycosis tends to infiltrate the orbit either directly through the superomedial wall from the maxillary sinus or posteriorly through the orbital apex of the nasolacrimal duct. There is infiltration of the retro-orbital fat which appears hypointense on the T1 W sequence and hyperintense on the T2W sequence. There is associated fat stranding with proptosis. Also, there is infiltration of the extraocular muscles which get bulky and show post-contrast enhancement (Fig. 5). There can be involvement of the preseptal soft tissue as well. When there is soft tissue at the orbital apex, it can present as orbital apex syndrome and has to be differentiated from its close differential diagnosis like chronic granulomatous diseases [13]. Rarely, endophthalmitis has been documented with fungal sinusitis [14]. Another rare complication is optic nerve infarction.

**Fig. 6:**
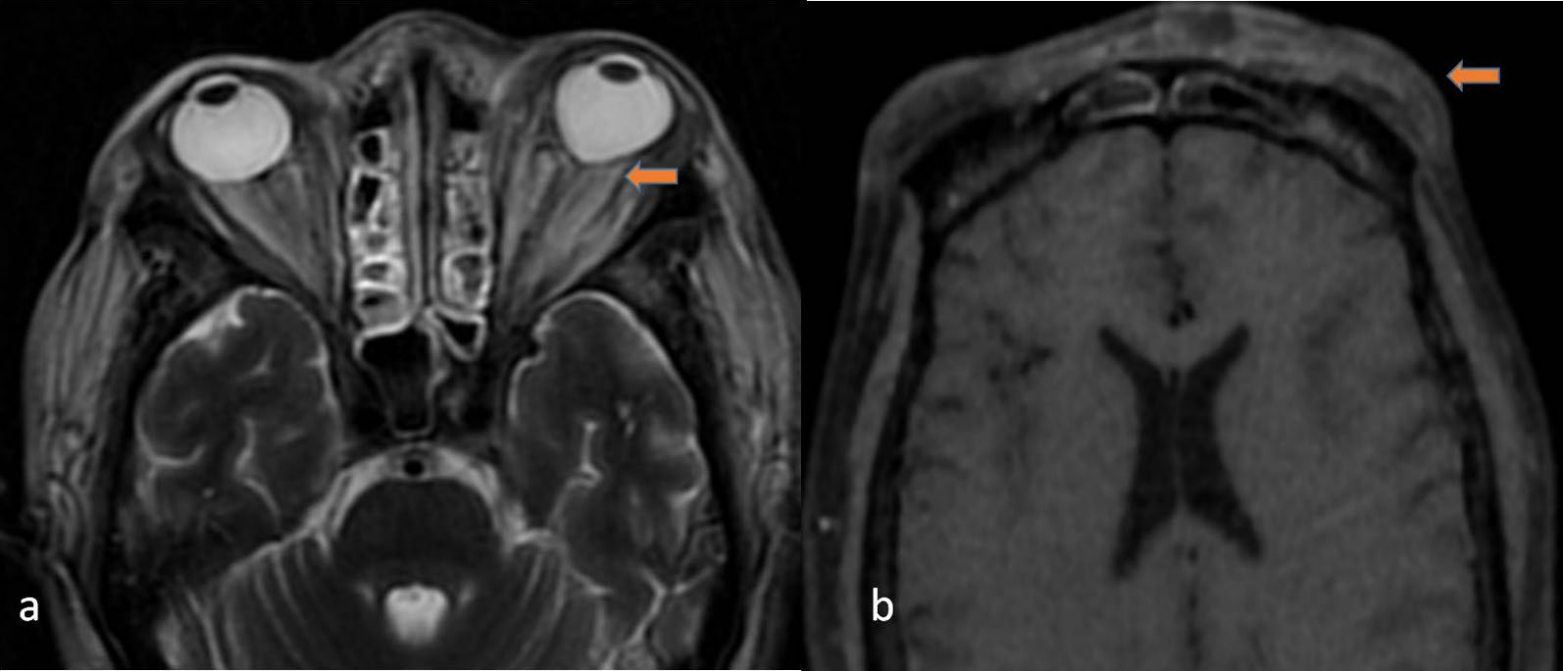
A 38-year-old female with a history of COVID infection 2 months back presented with nasal discharge, headache, and diminishing vision on the left side. Axial T2W image (**a**) showing proptosis on the left side with tenting of the posterior part of the globe *“guitar pick sign*”. Note the thinned-out left optic nerve. T1 W axial post-contrast image (**b**) showing left-sided preseptal soft tissue thickening

Due to proptosis there is stretching of the optic nerve which adheres to the posterior globe causing tenting of the posterior globe known as the “guitar pick sign” (Fig. 6). This sign can also be seen in other inflammatory pathologies or even in orbital trauma. Proven cases of mucormycosis in such cases showing positive guitar pick sign require extensive intravenous antifungal drugs and aggressive surgical debridement [15]. This sign when seen is an indication of prompt treatment as it can cause acute and permanent visual damage.

## Spread to the cavernous sinus

**Fig. 7:**
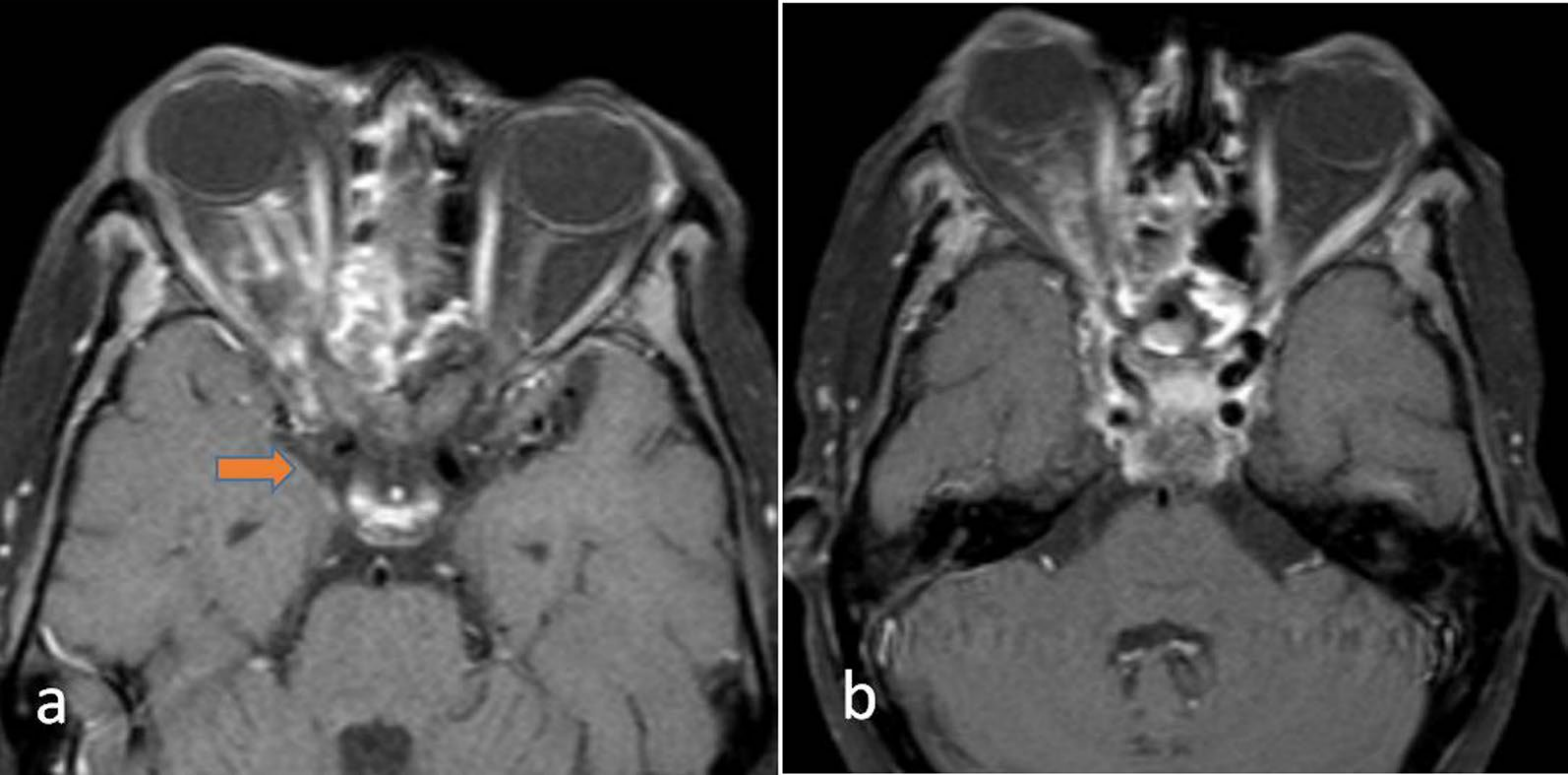
A 60-year-old diabetic male diagnosed case of Mucormycosis. Fat saturated axial T1W contrast-enhanced images (**a**, **b**) of showing right ethmoid sinusitis with right orbital extension. There is proptosis of the right globe, orbital fat stranding, avidly enhancing optic nerve sheath with the extension of enhancing soft tissue intracranially through the orbital apex(orange arrow), into right cavernous sinus with attenuation of right intracavernous ICA

**Fig. 8:**
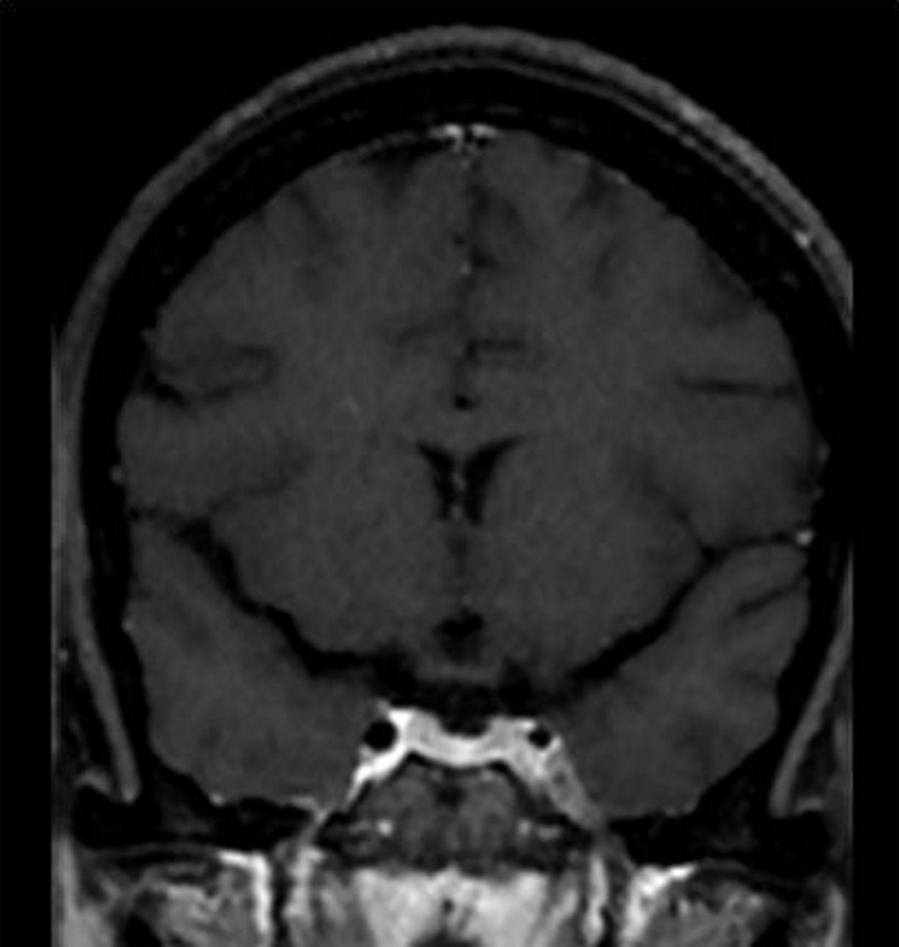
A 52-year-old diagnosed case of mucormycosis post-COVID infection. Coronal fat sat T1W post-contrast image showing the expansion of left cavernous sinus with non-enhancing area suggestive of partial thrombosis (orange arrow). Note the attenuated caliber of intracavernous left ICA

**Fig. 9:**
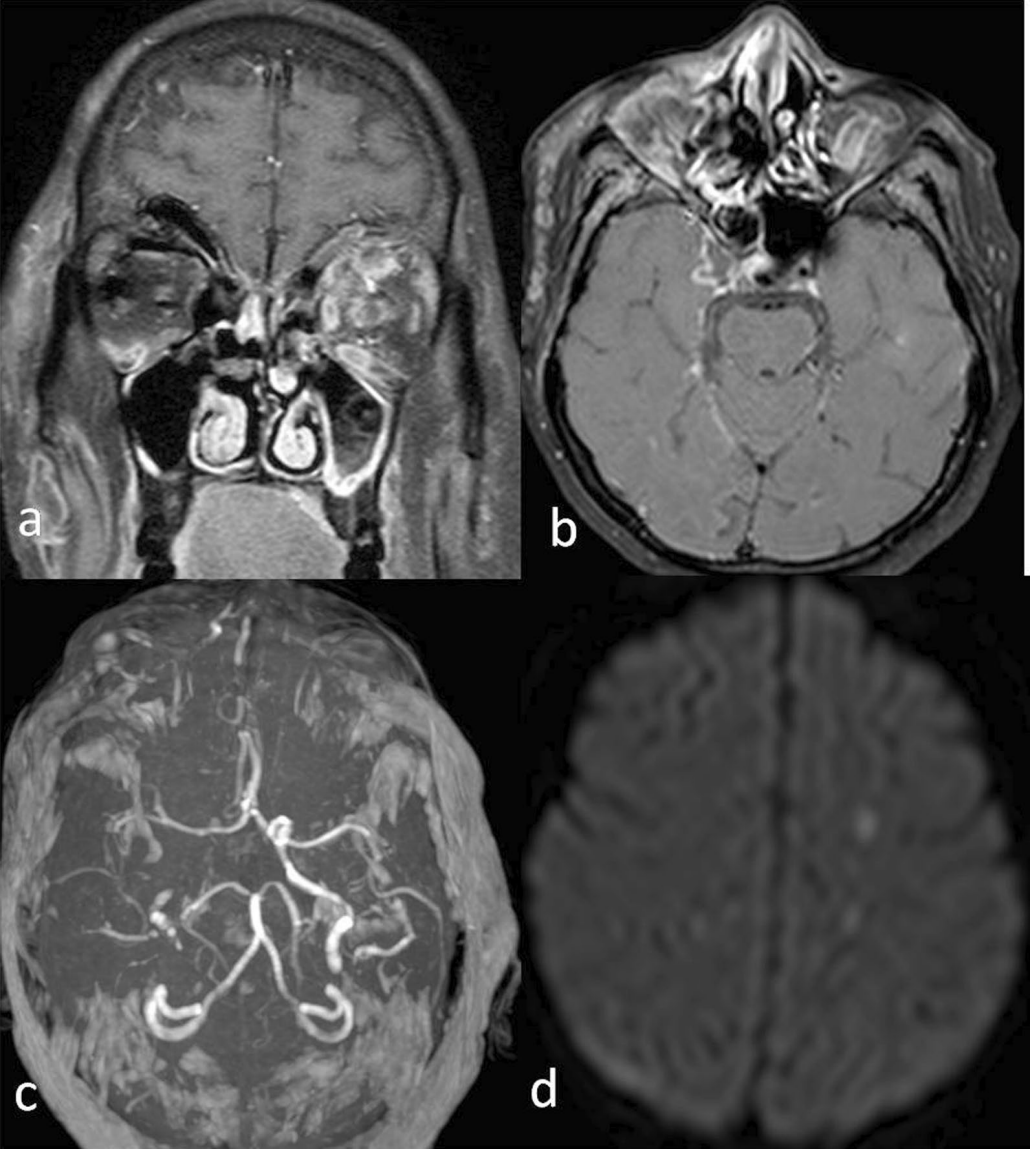
A 50-year-old diabetic patient with a history of COVID infection 1 month back, presented with acute onset nasal blockage and discharge, swelling on the right side of the face. Post-contrast T1 W coronal (**a**) and axial (**b**) images showing mucosal thickening of bilateral maxillary sinuses with contents in left maxillary sinus with the black turbinate sign of right middle turbinate. B/l Intraorbital extension of infection seen with left optic neuritis. Right cavernous sinus invasion is seen with loss of signal void of right intracavernous ICA. MIP images of MRA (**c**) show right ICA thrombosis. (**d**) DWI image showing few septic infarcts in left centrum semiovale. Findings were consistent with Invasive mucormycosis

The first intracranial structure to be involved is the cavernous sinus. Thrombosis of the cavernous sinus will involve the traversing cranial nerves, i.e. oculomotor, trochlear, abducens, ophthalmic and maxillary branches of the trigeminal nerve. It may also cause thrombosis of the internal carotid artery(ICA). On CT or MRI filling defects within the cavernous sinus appreciated as non-enhancing areas is a sign of cavernous sinus invasion (Figs. 7, 8). Further involvement of ICA will be seen as a lack of flow void of ICA on MRI (Fig. 9). Multifocal infarcts may be seen in involved ICA territory. Few recent case reports described unilateral occlusion of ICA with a rapid aggressive course of mucor mycosis [16].

## The central nervous system (CNS) spread

The spread of mucormycosis to CNS is amongst the most severe manifestations of this fungal infection causing high mortality and residual functional damage. The spread is most commonly direct through the paranasal sinuses and orbit in about 70% of the cases. The rest of the cases can be infected through the hematogenous route or as an isolated CNS infection in intravenous drug abusers [17]. Patients may present with focal seizures, hemiparesis, focal deficit, or altered sensorium.

The intracranial spread can be direct from the nasosinal cavities, through the ophthalmic arteries or the cribriform plate to the frontal lobes, or the cavernous sinus by orbital apex [18] One of the recent case reports also suggests intracranial extension through the olfactory tracts [19]. Initially, there may be an extradural invasion of the mucormycosis resulting in enhancing soft tissue in the extradural space of basifrontal regions or medial temporal lobe. It will have an associated enhancement of the overlying dura.

**Fig. 10:**
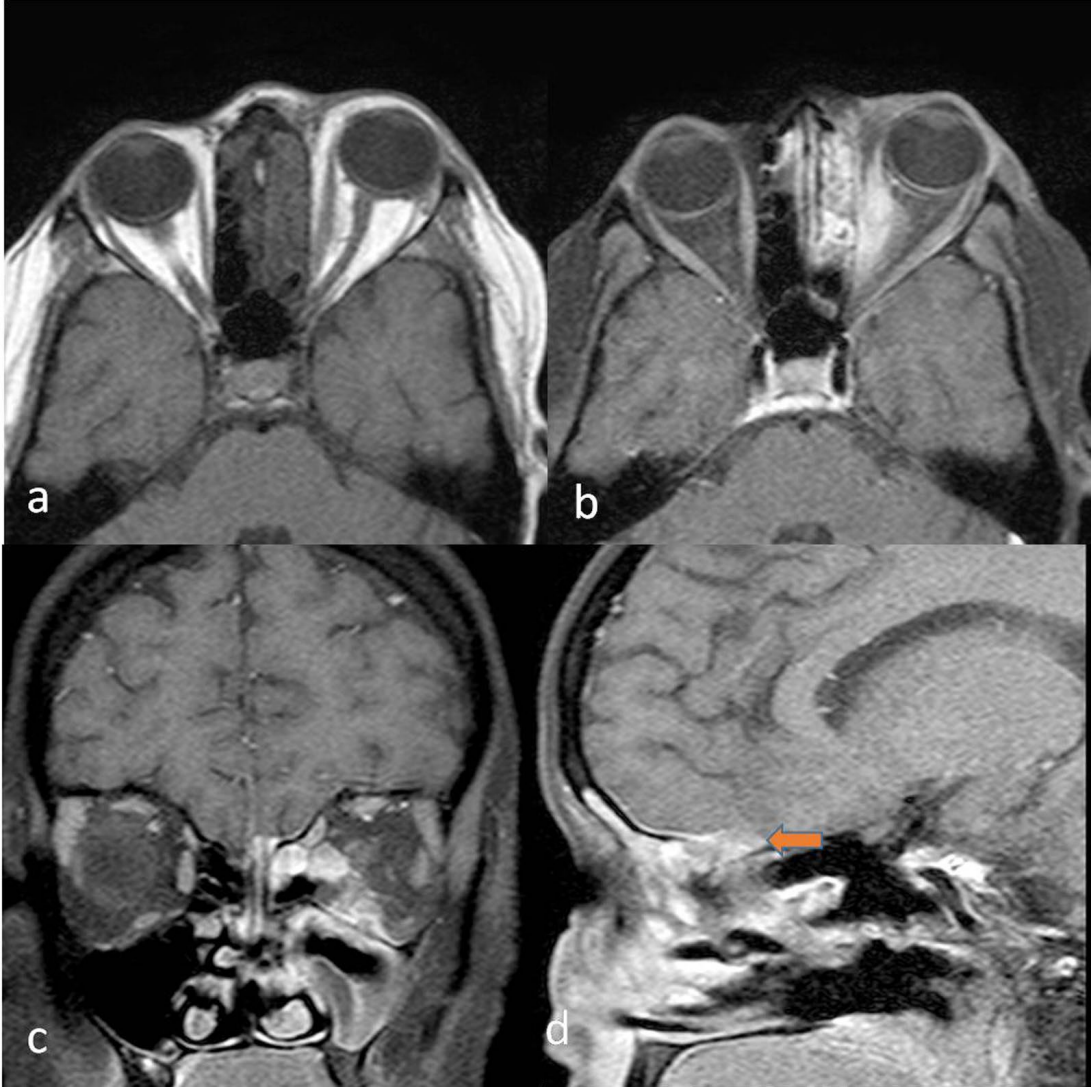
40-year-old male patient presented with complaints of left orbital and facial swelling with pain for the last one week. K/C/O ALL on maintenance phase chemotherapy. H/O COVID infection 7 months back. T1W axial (**a**), T1W fat-saturated post-contrast (**b**) images showing bilateral ethmoid sinusitis, bulky left medial rectus muscle with left-sided proptosis. There is enhancing thickening of the left preseptal soft tissue. T1W post-contrast coronal (**c**) and sagittal (**d**) images show myositis of left medial, inferior rectus, and superior oblique muscles along with spread along with left olfactory bulb left inferior orbital nerve and the cribriform plate with associated dural enhancement and subtle intraparenchymal invasion (arrow) in the left frontal region

As the infection spreads further, there may be a parenchymal invasion of the brain which will appear as T1 hypointense and T2 iso to mildly hyperintense soft tissue with diffusion restriction and post gadolinium enhancement (Fig. 10). Few blooming foci may also be appreciated.

There can be fungal abscesses with an irregular inner wall and T2 W hypointense contents. There shall be peripheral diffusion restriction due to the presence of fungal hyphae. Marked surrounding edema with mass effect may be seen. Trehalose peak on MR spectroscopy further authenticates the diagnosis of a fungal abscess [20].

At times, the fungal infiltration into the cerebral parenchyma may mimic a brain tumor [21] having a mass effect with increased cerebral blood volume and may require a histopathological diagnosis for confirmation.

Isolated involvement of the CNS is highly uncommon. This is seen in a few case reports in patients of intravenous drug abuse [22].

As the fungus is angioinvasive, it infests the blood vessels at the base of the brain resulting in infarction of the deep grey matter like basal ganglia [23]. Initially, it is unilateral with rapid progression to the other side basal ganglia. Cerebro Spinal fluid (CSF) examination does not yield the fungus in 50% of the cases and thus a high index of suspicion is advisable when isolated basal ganglia involvement is seen in intravenous drug abusers [24].

**Fig. 11:**
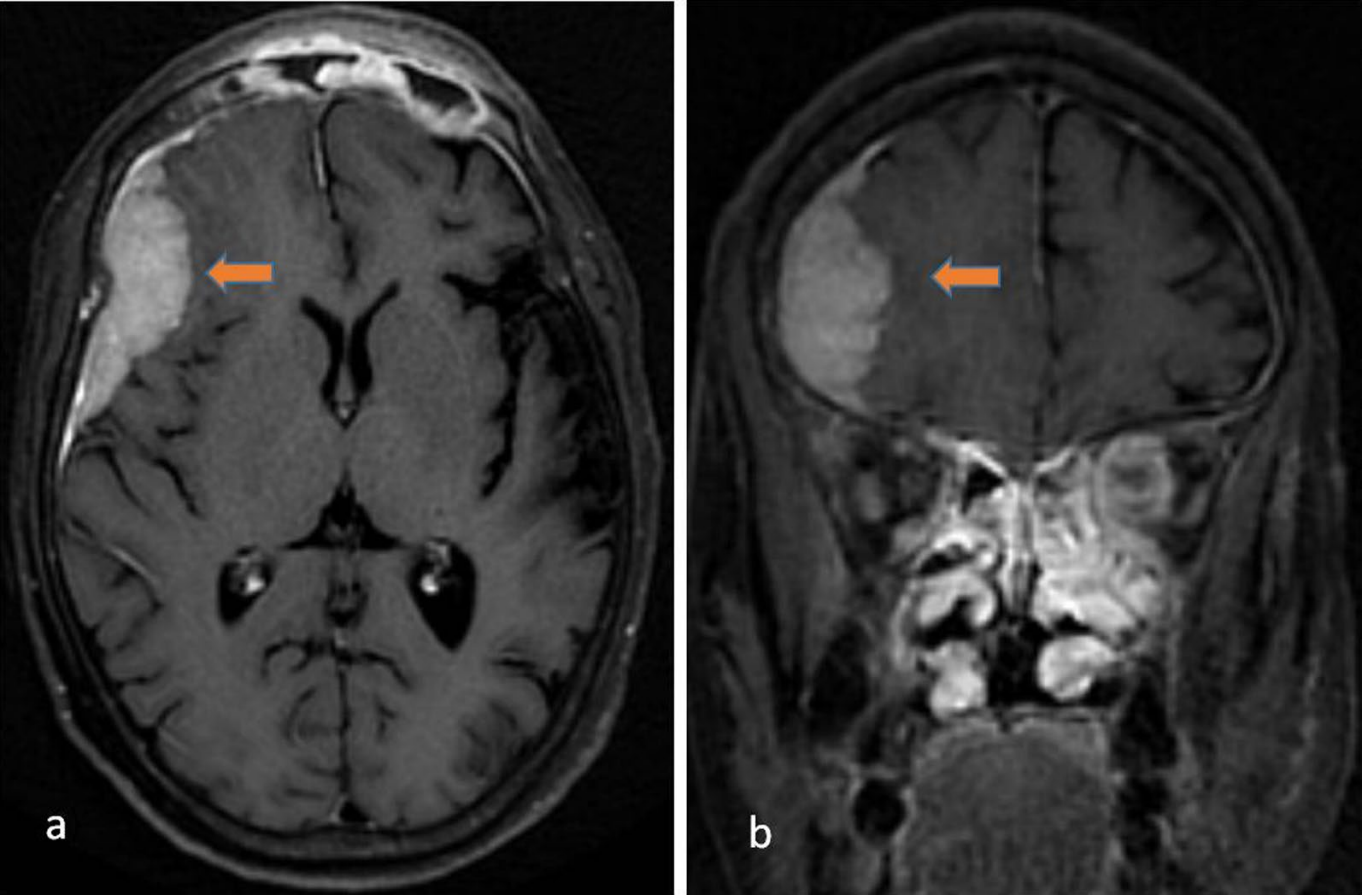
A 52-year-old female has a history of COVID infection 1 month back presented with headache and episodes of loss of consciousness. Contrast-enhanced T1W axial (**a**) and coronal (**b**) images in a patient of Sinonaso- orbital mucormycosis showing enhancing mass-like thickening of the dura in the right frontal region suggestive of hypertrophic pachymeningitis

In advanced disease, there may be a leptomeningeal enhancement. Rarely there may also be excessive thickening of the dura appearing as T2 iso to hypointense thickening with enhancement on post gadolinium images. This may be confused with meningioma. However, an appropriate clinical setting with regression on initiation of antifungal would support the diagnosis of fungal pachymeningeal hypertrophy [25] (Fig. 11).

Less commonly, there may be involvement of the bones of the calvarium resulting in osteomyelitis. On CT, this will appear as expansion, sclerosis, erosion, and irregular lytic lesions of the bone [26].

## Guidelines for radiologist

In any suspected case of Mucormycosis, the prime role of a radiologist is to assess the signs of fungal rhino-sinusitis, detect complications, if any, and define the exact extent of involvement to aid in surgical debridement. We have proposed a checklist for reporting a case of Mucormycosis (Table 1), which will allow for uniform reporting and aid the radiologist to form a comprehensive report.

**Table 1:** Reporting checklist for mucormycosis

1. Sino-nasal system	Mucosal thickening with variable enhancement with sinus wall
	Black turbinate sign
	Osteomyelitis or frank destruction
	Hyperdense contents in sinus
	Peri antral fat obliteration
2. Face	Signs of extensive cellulitis or collections in infratemporal fosse/pterygopalatine fosse, masticator space
3. Orbit	Extraconal – subperiosteal abscess, soft tissue fat stranding
	Conal- myositis, muscle infarctions
	Intraconal- collections, cellulitis, psedotumor
	Optic nerve- perineuritis, neuritis, infarction
	Orbital apex and superior orbital fissure
	Guitar pick sign
	Endophthalmitis
4. Cavernous sinus	Filling defect in the cavernous sinus
	Nerve enhancement
	ICA thrombosis
5. Intracranial	Extraaxial: meninigits, extradural/subdural epyema
	Cerebral: Abscess, direct invasion, septic embolic infarcts, large territorial ischemic infarcts
	Hypertrophic pachymeningitis
	Skull base osteomyelitis

## Conclusion

Mucormycosis is a recently emerging, often fatal fungal infection during the COVID-19 pandemic. A high index of suspicion of Mucormycosis must be kept in post-COVID cases presenting with sinusitis. Black turbinate sign in the nasal cavity, mucosal thickening in paranasal sinuses with periantral invasion, and bony erosion are the early diagnostic signs on imaging. As Mucormycosis is a locally aggressive opportunistic infection, close attention must be paid to define the exact extent of the disease, look for the spread to orbit and brain, detect complications like cavernous sinus thrombosis and ICA thrombosis. MRI should be performed in suspected cases to look for extra sinus extension which has high specificity for Mucormycosis. CT scan can be performed as a second-line modality. Knowledge of the entire imaging spectrum of Mucormycosis is extremely essential for formulating early diagnosis to reduce the associated morbidity and mortality.

## Data Availability

Not applicable.

## Abbreviations

CT: Computed tomography
MRI: Magnetic resonance imaging
W: Weighted
ICA: Internal carotid artery
